# A Molecular Study on the Prevalence and Virulence Potential of *Aeromonas* spp. Recovered from Patients Suffering from Diarrhea in Israel

**DOI:** 10.1371/journal.pone.0030070

**Published:** 2012-02-15

**Authors:** Yigal Senderovich, Shifra Ken-Dror, Irina Vainblat, Dvora Blau, Ido Izhaki, Malka Halpern

**Affiliations:** 1 Department of Evolutionary and Environmental Biology, Faculty of Natural Sciences, University of Haifa, Mount Carmel, Haifa, Israel; 2 Microbiology Laboratory of Clalit Health Services in Haifa, Haifa, Israel; 3 Department of Biology and Environment, Faculty of Natural Sciences, University of Haifa, Oranim, Tivon, Israel; The University of Hong Kong, China

## Abstract

**Background:**

Species of the genus *Aeromonas* are native inhabitants of aquatic environments and have recently been considered emerging human pathogens. Although the gastrointestinal tract is by far the most common anatomic site from which aeromonads are recovered, their role as etiologic agents of bacterial diarrhea is still disputed. *Aeromonas*-associated diarrhea is a phenomenon occurring worldwide; however, the exact prevalence of *Aeromonas* infections on a global scale is unknown.

**Methodology/Principal Findings:**

The prevalence and virulence potential of *Aeromonas* in patients suffering from diarrhea in Israel was studied using molecular methods. 1,033 diarrheal stools were sampled between April and September 2010 and *Aeromonas* species were identified in 17 (∼2%) patients by sequencing the *rpoD* gene. *Aeromonas* species identity and abundance was: *A. caviae* (65%), *A. veronii* (29%) and *Aeromonas taiwanensis* (6%). This is the first clinical record of *A. taiwanensis* as a diarrheal causative since its recent discovery from a wound infection in a patient in Taiwan. Most of the patients (77%) from which *Aeromonas* species were isolated were negative for any other pathogens. The patients ranged from 1 to 92 years in age. *Aeromonas* isolates were found to possess different virulence-associated genes: *ahpB* (88%), *pla/lip/lipH3/apl-1* (71%), *act/hlyA/aerA* (35%), *alt* (18%), *ast* (6%), *fla* (65%), *lafA* (41%), TTSS *ascV* (12%), TTSS *ascF-ascG* (12%), TTSS-dependent ADP-ribosylating toxins *aexU* (41%) and *aexT* (6%) in various combinations. Most of the identified strains were resistant to beta-lactam antibiotics but susceptible to third-generation cephalosporin antibiotics.

**Conclusions:**

*Aeromonas* may be a causative agent of diarrhea in patients in Israel and therefore should be included in routine bacteriological screenings.

## Introduction

*Aeromonas* species are waterborne, Gram-negative, oxidase-positive, rod-shaped bacteria that are ubiquitous in water. This includes chlorinated drinking water, as the bacteria can grow and survive in biofilms in the water distribution systems [1]. The prevalence of *Aeromonas* species in the aquatic environment has been recognized as a potential health risk, and some countries have adopted aeromonad counts as an additional indicator of water quality [2].

The most common clinical manifestations of *Aeromonas* infections are diarrhea, bacteremia and localized soft-tissue infections [3]. Patients may acquire *Aeromonas* infections both in community and hospital settings [3]–[5]. Both immunocompetent and immunocompromised patients are susceptible to *Aeromonas* infections [3], [6]. The gastrointestinal tract is by far the most common anatomic site from which aeromonads are recovered [3], [6]. *Aeromonas* have been isolated from children with acute diarrhea and from adults with traveler's diarrhea [3], [6]–[8]. The following species are frequently associated with diarrhea in humans: *A. hydrophila*, *A. veronii* bv. *sobria* and *A. cavia* [3], [6], [9], [10], [11].

The mechanism of *Aeromonas* pathogenesis is complex and not well understood. *Aeromonas* virulence is considered to be multifactorial. The virulence factors that were associated with *Aeromonas* pathogenicity are: cytotoxic enterotoxin, haemolysins, proteases [serine protease (*aspA*), elastase (*ahpB*)], lipases (*pla* and *plc*, *sat*), DNAses and adhesins [type IV pili and polar flagella (*flaA* and *flaB*)] [1], [6], [9], [10], [12], [13]. Several of these virulence factors have been identified in strains isolated from water [1]. In addition, genes for a type III secretion system (TTSS) were identified in this genus [14], [15]. TTSS has a role in delivering toxins directly into the host cell and in inducing apoptosis [14], [15].

In Israel, diarrhea patients are tested routinely by clinical laboratories for the presence of several bacterial pathogens, such as: *Campylobacter* spp., *Shigella* spp. and *Salmonella* spp., but not for *Aeromonas*. The aim of this research was to study the prevalence and virulence potential of *Aeromonas* spp. in diarrheal stools in Israel.

## Materials and Methods

### Ethics Statement

N/A. The data was analyzed anonymously.

We have applied to the ethics committee at Carmel Hospital, Clalit Health Services, Haifa, Israel, and the committee stated that such a research does not fall under the scope of the Helsinki Committee.

### *Aeromonas* Prevalence in Diarrheal Stools

The presence of *Aeromonas* was monitored in fecal specimens from diarrheal patients submitted to the Microbiology Laboratory of Clalit Health Services in Haifa. This Laboratory provides services to a wide range of population, from the district of Haifa and West Galilee in Israel (this is a community health service, not a hospital). The surveillance was conducted between April 13 and September 15, 2010 (five months). All specimens were checked routinely for the following enteropathogens: *Shigella*, *Salmonella* and *Campylobacter* spp. were isolated and identified by conventional methods [16]; *Rotavirus* was detected by an antigen detection method (Novamed, Israel); parasites were studied according to methods described in Garcia and Isenberg [17]. For the isolation of *Aeromonas* spp. the fecal specimens were either enriched in alkaline peptone water (APW) containing peptone (1%, wt/vol) and NaCl (1%, wt/vol) pH 8.5, or directly streaked on a selective m-Aeromonas agar base (Havelaar Biolife, Milano, Italy). In the case of enrichment, the tubes were incubated at 37°C without shaking for 6–18 h, and then streaked on m-Aeromonas selective agar. The agar plates were incubated overnight at 37°C. Colonies that were morphologically suspected as *Aeromonas* (yellow, smooth and rounded) were subcultured onto LB agar (Himedia, India), and then tested for oxidase (1% tetramethyl-phenylenediamine, Sigma). The identity of the isolates with positive results was further verified by *Aeromonas* genus specific PCR assay in accordance with Kong et al. (1999) [18]. Reddy Mix PCR master mixture (ABgene, Epsom, UK) was used for the DNA amplification. All the isolates that were found to belong to the *Aeromonas* genus were maintained in LB with 30% glycerol (−80°C).

*Aeromonas* isolates were further identified by amplifying and sequencing the housekeeping gene *rpoD*, encoding *σ* ^70^ factor, which is one of the sigma factors that confer promoter-specific transcription initiation on RNA polymerase [19]. The PCR products were sequenced by MCLAB (San Francisco, CA). Newly determined sequences were compared to those available in the GenBank database, using the standard nucleotide–nucleotide BLAST program (BLASTN; http://www.ncbi.nlm.nih.gov), to ascertain their closest relatives. The sequences were submitted to the GenBank database under accession numbers JF738005–JF738021. A phylogenetic tree was generated using the neighbor-joining method with NJPlot (MEGA 4.1) based on alignments from CLUSTAL W.

### Virulence Factors and Antimicrobial Susceptibility

The presence of the following genes encoding virulence factors was determined in all *Aeromonas* isolates: cytotoxic enterotoxin (*act*)/aerolysin (*aerA*)/haemolysin (*hlyA*) by using one set of primers AHCF1/AHCR1 [12]; *alt* and *ast* genes for cytotonic enterotoxins; *ahyB* gene for elastase; *pla*/*lipH3*/*apl-1*/*lip* genes for phospholipase; and *fla* gene for flagellin [1]. The presence of the genes *act*/*aerA*/*hlyA* and *ast*; *fla* and *alt*; *ahyB* and *pla*/*lipH3*/*apl-1*/*lip* was tested simultaneously in the same reaction mixture, in accordance with Sen and Rodgers (2004) [1]. The presence of genes encoding the components of the type III secretion system, *ascV*, *ascF-ascG* [15], type III secretion dependent ADP-ribosylating toxins, *aexT* and *aexU* [20], and of *lafA* gene encoding a lateral flagella [13] was determined as well.

The disk diffusion antimicrobial susceptibility tests were performed by a standardized method [21], [22]. All disks were purchased from OXOID (UK).

## Results

### *Aeromonas* Prevalence in Diarrheal Stools

**Figure 1:**
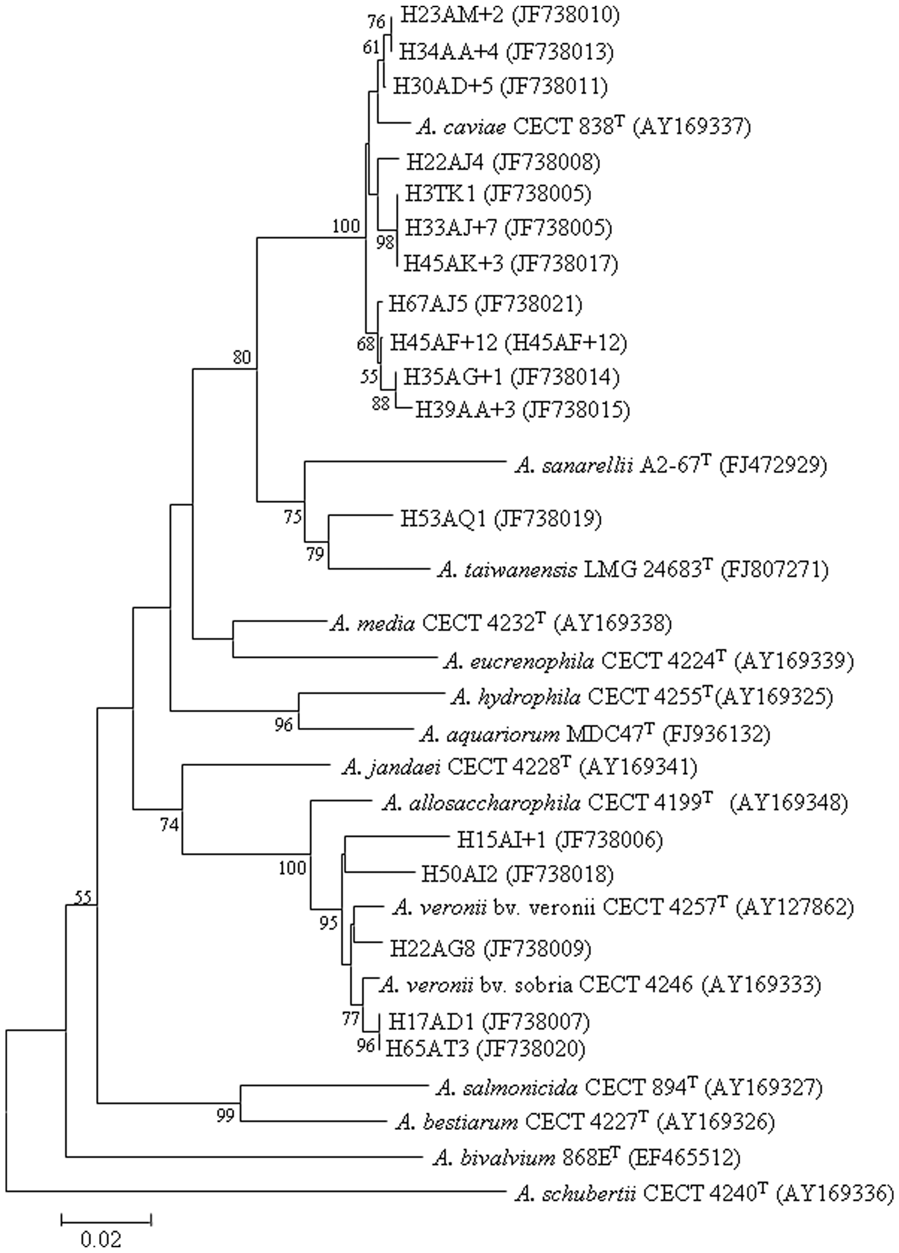
Phylogenetic tree of *Aeromonas* isolates recovered from diarrhea patients. The tree shows the relationships based on partial sequences of *rpoD* gene of type strains of *Aeromonas* species and the isolates from the current study. The sequence alignments were performed using the CLUSTAL W program, and the tree was generated using the neighbor–joining method with Kimura 2 parameter distances in MEGA 4.1 software. Bootstrap values (from 1,000 replicates) greater than 50% are shown at the branch points. The bar indicates 2% sequence divergence.

A total of 1,033 stool specimens from patients suffering from diarrhea were monitored for the presence of *Aeromonas* during a five month period between April 13 and September 15, 2010. Seventeen patients (∼2%) tested positive for *Aeromonas* species which included 11 (65%) *A. caviae*, five (29%) *A. veronii*, and one strain, (H53AQ1). This strain showed the highest *rpoD* gene similarity (96%) to the deposited sequence of the type strain of *A. taiwanensis*, and clustered with this species in the phylogenetic tree (Figure 1).

The specimens of the diarrhea patients were also checked for other enteropathogenes. The results revealed that pathogenic bacteria, *Rotavirus* and parasites were recovered from about 15% (155 of 1033) of the diarrhea patients. The prevalence of the detected enterophathogenes was; *Campylobacter* sp. 5.2%, *Shigella* 3.3%, *Salmonella enterica* 2%, *Aeromonas* spp. 2%, *Rotavirus* 0.4%, *Giardia lamblia* 2.3%, and *Cryptosporidium parvum* 0.15%. Mixed infections were found in four patients that were positive for *Aeromonas* as well as for other known enteropathogens (Table S1).

### Virulence Factors and Antimicrobial Susceptibility

All *Aeromonas* isolates were screened for the presence of virulence genes (Table 1). The most prevalent genes were *ahyB* (88%) and *pla*/*lipH3*/*apl-1*/*lip* (71%). The two types of flagella that were screened (polar and lateral) were quite prevalent as well (65% and 41%, respectively). In every strain that was positive for the genes encoding the TTSS, a gene for the effector *aexU* was present as well. The *ast* gene was found only in one isolate (H65AT3), which was identified as *A. veronii*. The virulence genotypes were found in different combinations: three isolates (18%) possessed five different genes, four (24%) possessed three or four different genes and two (12%) possessed two different genes (Table 1).

**Table 1:** Prevalence of virulence genes in *Aeromonas* isolates from diarrheal patients.

Isolatename	Virulence genes										
	*ahpB*	*pla/lip/lipH3/apl-1*	*act/aerA/hlyA*	*ast*	*alt*	*fla*	*lafA*	TTSS*ascV*	TTSS*ascF-ascG*	*aexT*	*aexU*
H3TK1	+	+	−	−	−	+	−	−	−	−	−
H15AI+1	+	+	+	−	+	−	−	−	−	−	−
H17AD1	−	−	+	−	+	+	−	−	−	+	−
H22AJ4	+	+	−	−	−	−	−	−	−	−	−
H22AG8	−	−	+	−	−	+	−	+	+	−	+
H23AM+2	+	+	+	−	−	+	+	−	−	−	−
H30AD+5	+	+	−	−	−	−	+	−	−	−	−
H33AJ+7	+	−	−	−	−	+	−	−	−	−	+
H34AA+4	+	+	+	−	−	+	+	−	−	−	−
H35AG+1	+	+	−	−	−	+	+	−	−	−	−
H39AA+3	+	+	−	−	+	−	−	−	−	−	+
H45AF+12	+	+	−	−	−	+	+	−	−	−	+
H45AK+3	+	+	−	−	−	+	−	−	−	−	−
H50AI2	+	+	+	−	−	+	−	+	−	−	+
H53AQ1	+	−	−	−	−	+	−	−	+	−	+
H65AT3	−	+	−	+	−	−	+	−	−	−	−
H67AJ5	+	+	−	−	−	−	+	−	−	−	+

For more details on the isolates and on the patients see Table S1.

The susceptibility of *Aeromonas* isolates was evaluated against 15 antimicrobial agents. All isolates were susceptible to amikacin, cefotaxime, ceftazidime, ceftriaxone, ciprofloxacin and chloramphenicol; however, they varied in their susceptibility to other antimicrobial agents (Table 2). The three *Aeromonas* species displayed the same antibiotic sensitivity patterns.

**Table 2:** Susceptibility of *Aeromonas* isolates to antimicrobial agents.

Antimicrobial agent		Number (%) of strains	
(number of strains tested)	susceptible	intermediate resistance	resistant
amikacin (11)	11 (100)	-	-
cefotaxime (11)	11 (100)	-	-
ceftazidime (11)	11 (100)	-	-
ceftriaxone (E test) (11)	11 (100)	-	-
ciprofloxacin (11)	11 (100)	-	-
chloramphenicol (11)	11 (100)	-	-
gentamicin (11)	10 (91)	-	1 (9)
piperacillin–tazobactam (11)	10 (91)	-	1 (9)
trimethoprim–sulfamethoxazole (11)	10 (91)	-	1 (9)
imipenem (also meropenem) (11)	8 (73)	-	3 (27)
cefoxitin (10)	7 (70)	2 (20)	1 (10)
nalidixic acid (11)	7 (64)	-	4 (36)
tetracycline (11)	6 (55)	-	5 (45)
amoxicillin+clavulanic acid (11)	3 (27)	3 (27)	5 (46)
cephalotin (10)	2 (20)	-	8 (80)

Most of the identified strains were resistant to beta-lactam antibiotics but susceptible to third-generation cephalosporin antibiotics.

## Discussion

Despite the existence of detailed case reports and epidemiological case control investigations, the role of *Aeromonas* as the etiological agent of bacterial diarrhea has been questioned and debated several times [3], [6], [11], . Figueras et al. [26] rebutted arguments provided by several authors against considering *Aeromonas* a true enteropathogenic bacterium one by one. Today it is well accepted that if *Aeromonas* can cause different infections like cellulitis, meningitis, pneumonia, wound infections and more in healthy humans, it can also have the capacity to produce diarrhea [3], [6], [26].

In several reported studies throughout the world, *Aeromonas* species have been isolated at a rate of 0.6 to 7.2% in patients with diarrhea, predominantly in infants and children [2], [27]. In the current study, *Aeromonas* positive patients ranged in age and only two out of 17 isolates were taken from children (Table S1). The current study relied on a limited amount of strains as it was performed only during a period of five months; however, the *Aeromonas* isolation rate amounted to about 2%, which is similar to the rate obtained in other studies performed in other countries [3,6 and references therein], as well as in Israel in 1990 [28]. In a recent study performed by Pablos et al. [29] in León (Spain) they found a frequency of *Aeromonas* of 4% (32 positive patients of the 800 investigated), mainly associated with infant or pediatric patients (68.8%). Furthermore they found mixed infections with other pathogens in 12 patients [29]. In our study, only two of the patients were infants (12%), and all four patients that had mixed infections were adults (Table S1).

In mixed infections, *Aeromonas* may be transient colonizers lacking a causal relationship with a disease, but in some cases, multiple pathogens may act synergistically to produce diarrhea [30]. *Aeromonas* species are carried asymptomatically by some individuals [3], [6] as occurs with other recognized enteropathogens like *Salmonella*. However, a study that was performed in 1990 in Israel compared the prevalence of *Aeromonas* in the stools obtained from 932 adult patients with acute diarrhea (recovered between 1986 and 1987) to 500 stools from asymptomatic controls. They found an *Aeromonas* prevalence of about 2% in the diarrhea cases, which conforms to our study. But no *Aeromonas* were detected in the controls [28]. This seems to indicate a clear association of *Aeromonas* with diarrhea cases in Israel, as we found in our study.

Among the recognized *Aeromonas* species, *A. veronii* bv. *sobria*, *A. caviae* and *A. hydrophila* are more frequently associated with diarrhea in humans, representing 85% of clinical isolates [11]. Interestingly, none of the identified strains in the current study belonged to *A. hydrophila*. This is in agreement with the false importance attributed to this species on the basis of phenotypic identifications [3], [26]. In the current study, *A. caviae* was the predominating species (65%, 11/17), followed by *A. veronii* that was isolated from five patients (29%). One patient carried a strain that was identified as *A. taiwanensis* (Figure 1). All the strains were identified using the *rpoD* gene sequencing method. *Aeromonas* identification on the basis of *rpoD* gene sequencing is considered to be much more accurate than 16S rRNA gene sequencing or biochemical identification methods. The fact that many studies found *A. hydrophila* a major species to cause diarrhea (among *Aeromonas* species) may be due to limitations in the identification methods that were used in those studies [3], [26].

The current study provides the first clinical record of *A. taiwanensis* as a diarrheal causative since this species was identified [31]. So far, the only available strain (the type strain) was recovered from an infected burn wound of a 40 years old male [31] and in the current study the strain was isolated from feces of a 35 years old diarrheal female patient.

The clinical manifestations of *Aeromonas* associated gastroenteritis can range from mild self-limiting watery diarrhea to a more severe and invasive dysenteric form. Chronic diarrhea episodes and isolated cases of a cholera-like illness have also been described [11]. The bacterial flagella are thought to play an important role in pathogenicity. *Aeromonas* produces two types of flagella: a constitutively expressed polar flagellum (*fla*) and multiple inducible lateral flagella (*laf*). Both types play a role in the attachment of the bacteria to the gastrointestinal epithelium, biofilm formation and long-term colonization [6]. Both types of flagella (*fla* and *lafA*) were common among the *Aeromonas* isolates from the patients in the current study (Table 1). The occurrence of genes encoding hemolytic, cytotonic, cytotoxic, and enterotoxic activities (*aerA*, *hlyA*, *alt*, *ast*, *act*) may contribute to diarrheal-related virulence [6], [31], [32]. In the present study, 35% of the *Aeromonas* isolates possessed the *act/aerA/hlyA* gene. The most prevalent virulence-associated genes in the isolates from our study were *ahpB* for elastase (88%) and *pla/lip/lipH3/apl-1* for lipase (71%) (Table 1). These genes may be essential for the ability of the bacterium to adhere and invade the intestinal mucosa [1].

Type III secretion system (TTSS) plays crucial roles in host-pathogen interactions [14], [15]. One of the best-described toxins that are translocated via a TTSS is the ADP-ribosylating toxin, AexT. This toxin was found to be more common among the environmental, rather than the clinical *Aeromonas* strains [13]. In our study, the gene for this toxin was detected only in one strain. Recently, a novel type-three-secretion-dependent effector, AexU, was discovered in *Aeromonas*. AexU is an ADP-ribosylating toxin and is required for virulence of *Aeromonas hydrophila* in mice [20]. The gene for this toxin was quite prevalent among the strains in our study (41%). The prevalence of the genes encoding TTSS apparatus (12%) was lower than the *aexU* gene prevalence (41%). The TTSS is probably underrepresented, as may happen in PCR based studies. Nevertheless, the presence of *aexU* gene strengthens the case of *Aeromonas* being recognized as a stronger pathogen.

In another study that surveyed the distribution of virulence associated genes among *Aeromonas* species from human stool specimens in Spain, it was found that *alt*, *ast*, *laf*, *aerA*, and *hlyA* genes were present in 72, 19, 3, 25, and 28% of the strains, respectively. None of the strains harbored *ascF – G* [29]. In clinical diarrheic isolates of *A. hydrophila* in Spain the distribution of associated virulence genes was different: *alt* – 82%, *ast* – 96%, *laf* – 77%, *aexT* – 5%, *ascV* – 5% [13].

*Aeromonas* species are known to be intrinsically susceptible to all antibiotics active against non-fastidious Gram-negative bacilli, except for many beta–lactams, due to the production of multiple inducible, chromosomally encoded β–lactamases [33]. In our study, most strains (80%) were resistant to cephalotin and partially resistant to amoxicillin combined with clavulanic acid (46%) (Table 2). All strains were susceptible to third-generation cephalosporin antibiotics (cefotaxime, ceftazidime, ceftriaxone), second-generation fluoroquinolone antibiotics – ciprofloxacin, aminoglycoside antibiotic – amikacin, and to chloramphenicol.

Recently, it was found that the egg masses of chironomids, non-biting midges, (*Diptera; Chironomidae*) serve as a natural reservoir for *Aeromonas* pathogenic species [34], [35] as well as for *Vibrio cholerae* [36]. Chironomid infestations in drinking water supply systems are an existing problem in Israel [37] and worldwide [38]. Chironomids may disseminate pathogenic species of *Aeromonas* between drinking water reservoirs, as was suggested for *V. cholerae* [39].

The source of *Aeromonas* in diarrheal patients was not investigated in the current study. In order to investigate the route of transmission of *Aeromonas* pathogenic strains an extensive study on strains from various origins should be performed. Chironomid egg masses in drinking water ponds and tap waters should be screened for *Aeromonas* isolates and compared with isolates from diarrheal patients.

*Aeromonas* infections are self–limiting, but their diagnosis may be crucial in young children, old and immunocompromised patients. We conclude that *Aeromonas* may be a causative agent of diarrhea in patients in Israel and therefore should be included in routine bacteriological screenings.

## Supporting Information

Table S1 **Characterization of** ***Aeromonas*** **isolates from diarrheal patients.** *Aeromonas rpoD* sequences were deposited in the GenBank database under the accession numbers JF738005–JF738021 (see also Figure 1).
(DOC)
