# Supplementary material for: A Molecular Study on the Prevalence and Virulence Potential of Aeromonas spp. Recovered from Patients Suffering from Diarrhea in Israel
Source: PLoS One. 2012 Feb 15;7(2):e30070. doi: 10.1371/journal.pone.0030070 (PMC3280246; doi:10.1371/journal.pone.0030070)
Supplement: Table S1 — Characterization of Aeromonas isolates from diarrheal patients. Aeromonas rpoD sequences were deposited in the GenBank database under the accession numbers JF738005–JF738021 (see also Figure 1). (DOC) [file pone.0030070.s001.doc]

**Supplementary Table**

**Table S1. Characterization of *Aeromonas* isolates from diarrheal patients.** *Aeromonas rpoD* sequences were deposited in the GenBank database under the accession numbers JF738005-JF738021 (see also Figure 1).

| **Isolate name** | **Species identification** | **Date of isolation**  **(2010)** | **Patient's age**  **and gender*** | **Mixed infection with another enteropathogen** |
| --- | --- | --- | --- | --- |
| H3TK1 | *A. caviae* | April, 13 | 92, M | *Salmonella enterica* |
| H15AI+1 | *A. veronii* | May, 10 | 23, M |  |
| H17AD1 | *A. veronii* | May, 31 | 61, M |  |
| H22AJ4 | *A. veronii* | June, 07 | 59, M |  |
| H22AG8 | *A. caviae* | June, 07 | 20, M | *Campylobacter jejuni* |
| H23AM+2 | *A. caviae* | June, 08 | 21, M |  |
| H30AD+5 | *A. caviae* | June, 22 | 23, F |  |
| H33AJ+7 | *A. caviae* | June, 28 | 27, M |  |
| H34AA+4 | *A. caviae* | June, 29 | 28, M |  |
| H35AG+1 | *A. caviae* | June, 30 | 26, F | *Shigella sonnei* |
| H39AA+3 | *A. caviae* | July, 11 | 65, M |  |
| H45AF+12 | *A. caviae* | Aug, 01 | 62, F |  |
| H45AK+3 | *A. caviae* | Aug, 01 | 1, F |  |
| H50AI2 | *A. veronii* | Aug, 16 | 5, M |  |
| H53AQ1 | *A*. *tatwanensis* | Aug, 22 | 35, F |  |
| H65AT3 | *A. veronii* | Sept, 13 | 59, M | *Clostridium difficile* |
| H67AJ5 | *A. caviae* | Sept, 15 | 69, F |  |

*M (male); F (female)
